# Risk factors associated with short-term adverse events after SARS-CoV-2 vaccination in patients with immune-mediated inflammatory diseases

**DOI:** 10.1186/s12916-022-02310-7

**Published:** 2022-03-02

**Authors:** Luuk Wieske, Laura Y. L. Kummer, Koos P. J. van Dam, Eileen W. Stalman, Anneke J. van der Kooi, Joost Raaphorst, Mark Löwenberg, R. Bart Takkenberg, Adriaan G. Volkers, Geert R. A. M. D’Haens, Sander W. Tas, Phyllis I. Spuls, Marcel W. Bekkenk, Annelie H. Musters, Nicoline F. Post, Angela L. Bosma, Marc L. Hilhorst, Yosta Vegting, Frederike J. Bemelman, Joep Killestein, Zoé L. E. van Kempen, Alexandre E. Voskuyl, Bo Broens, Agner Parra Sanchez, Gertjan Wolbink, Laura Boekel, Abraham Rutgers, Karina de Leeuw, Barbara Horváth, Jan J. G. M. Verschuuren, Annabel M. Ruiter, Lotte van Ouwerkerk, Diane van der Woude, Cornelia F. Allaart, Y. K. Onno Teng, Pieter van Paassen, Matthias H. Busch, B. Papay Jallah, Esther Brusse, Pieter A. van Doorn, Adája E. Baars, Dirkjan Hijnen, Corine R. G. Schreurs, W. Ludo van der Pol, H. Stephan Goedee, Maurice Steenhuis, Theo Rispens, Anja ten Brinke, Niels J. M. Verstegen, Koos A. H. Zwinderman, S. Marieke van Ham, Taco W. Kuijpers, Filip Eftimov, on behalf of the T2B! immunity against SARS-CoV-2 study group

**Affiliations:** 1 Department of Neurology and Neurophysiology, Amsterdam Neuroscience, Amsterdam UMC, Location Academic Medical Centre, University of Amsterdam, Amsterdam, The Netherlands; 2 Department of Clinical Neurophysiology, St Antonius Hospital, Nieuwegein, The Netherlands; 3 Department of Immunopathology, Sanquin Research and Landsteiner Laboratory, Amsterdam UMC, Amsterdam, The Netherlands; 4 Department of Gastroenterology and Hepathology, Amsterdam UMC, Location Academic Medical Center, University of Amsterdam, Amsterdam, The Netherlands; 5 Department of Rheumatology and Clinical Immunology, Amsterdam Rheumatology and Immunology Center, Amsterdam UMC, University of Amsterdam, Amsterdam, The Netherlands; 6 Department of Dermatology, Amsterdam UMC, Location Academic Medical Center, University of Amsterdam, Amsterdam, The Netherlands; 7 Amsterdam Institute for Public Health/Infection and Immunology, University of Amsterdam, Amsterdam, The Netherlands; 8 Department of Internal Medicine, Section of Nephrology, Amsterdam UMC, Location Academic Medical Center, University of Amsterdam, Amsterdam, The Netherlands; 9 Department of Neurology, Amsterdam UMC, VU University Medical Center, Amsterdam, The Netherlands; 10 Department of Rheumatology and Clinical Immunology, Amsterdam Rheumatology and Immunology Center, VU University Medical Center, Amsterdam, The Netherlands; 11 Department of Rheumatology, Amsterdam Rheumatology and Immunology Center, Location Reade, Amsterdam, The Netherlands; 12 Department of Rheumatology and Clinical Immunology, University Medical Center Groningen, Groningen, The Netherlands; 13 Department of Dermatology, Center for Blistering Diseases, University Medical Center Groningen, University Groningen, Groningen, The Netherlands; 14 Department of Neurology, Leiden University Medical Center, Leiden, The Netherlands; 15 Department of Rheumatology, Leiden University Medical Center, Leiden, The Netherlands; 16 Centre of Expertise for Lupus-, Vasculitis- and Complement-Mediated Systemic Diseases, Department of Internal Medicine – Nephrology Section, Leiden University Medical Centre, Leiden, The Netherlands; 17 Department of Nephrology and Clinical Immunology, Maastricht University Medical Center, Maastricht, The Netherlands; 18 Department of Neurology, Erasmus MC University Medical Center, Rotterdam, The Netherlands; 19 Department of Dermatology, Erasmus MC University Medical Center, Rotterdam, The Netherlands; 20 Department of Neurology and Neurosurgery, Brain Center UMC Utrecht, Utrecht, The Netherlands; 21 Clinical Research Unit, Amsterdam UMC, Location Academic Medical Centre, University of Amsterdam, Amsterdam, The Netherlands; 22 Swammerdam Institute for Life Sciences, University of Amsterdam, Amsterdam, The Netherlands; 23 Department of Pediatric Immunology, Rheumatology and Infectious Disease, Amsterdam UMC, Location Academic Medical Centre, University of Amsterdam, Amsterdam, The Netherlands

## Abstract

**Background:**

Studies have suggested incremental short-term adverse events (AE) after repeated vaccination. In this report, we assessed occurrence and risk factors for short-term AEs following repeated SARS-CoV-2 vaccination in patients with various immune-mediated inflammatory diseases (IMIDs).

**Methods:**

Self-reported daily questionnaires on AEs during the first 7 days after vaccination were obtained of 2259 individuals (2081 patients and 178 controls) participating in an ongoing prospective multicenter cohort study on SARS-CoV-2 vaccination in patients with various IMIDs in the Netherlands (T2B-COVID). Relative risks were calculated for potential risk factors associated with clinically relevant AE (rAE), defined as AE lasting longer than 2 days or impacting daily life.

**Results:**

In total, 5454 vaccinations were recorded (1737 first, 1992 second and 1478 third vaccinations). Multiple sclerosis, Crohn’s disease and rheumatoid arthritis were the largest disease groups. rAEs were reported by 57.3% (95% CI 54.8–59.8) of patients after the first vaccination, 61.5% (95% CI 59.2–63.7) after the second vaccination and 58% (95% CI 55.3–60.6) after the third vaccination. At day 7 after the first, second and third vaccination, respectively, 7.6% (95% CI 6.3–9.1), 7.4% (95% CI 6.2–8.7) and 6.8% (95% CI 5.4–8.3) of patients still reported AEs impacting daily life. Hospital admissions and allergic reactions were uncommon (<0.7%).

Female sex (aRR 1.43, 95% CI 1.32–1.56), age below 50 (aRR 1.14, 95% CI 1.06–1.23), a preceding SARS-CoV-2 infection (aRR 1.14, 95% CI 1.01–1.29) and having an IMID (aRR 1.16, 95% CI 1.01–1.34) were associated with increased risk of rAEs following a vaccination. Compared to the second vaccination, the first vaccination was associated with a lower risk of rAEs (aRR 0.92, 95% CI 0.84–0.99) while a third vaccination was not associated with increased risk on rAEs (aRR 0.93, 95% CI 0.84–1.02). BNT162b2 vaccines were associated with lower risk on rAEs compared to CX-024414 (aRR 0.86, 95% CI 0.80–0.93).

**Conclusions:**

A third SARS-CoV-2 vaccination was not associated with increased risk of rAEs in IMID patients compared to the second vaccination. Patients with an IMID have a modestly increased risk of rAEs after vaccination when compared to controls. Most AEs are resolved within 7 days; hospital admissions and allergic reactions were uncommon.

**Trial registration:**

NL74974.018.20, Trial ID: NL8900. Registered on 9 September 2020.

**Supplementary Information:**

The online version contains supplementary material available at 10.1186/s12916-022-02310-7.

## Background

Many countries are providing third SARS-CoV-2 vaccinations to patients with immune-mediated inflammatory diseases (IMIDs) using immunosuppressive agents due to a (suspected) insufficient immune response after a two-dose vaccination strategy [1, 2]. Reported incremental adverse events (AEs) in healthy individuals after the second vaccination give concerns [3–5]. However, large studies on incremental AEs after three SARS-CoV-2 vaccinations in patients with IMIDs are missing.

## Methods

We describe an interim analysis on short-term AEs following SARS-CoV-2 vaccinations of an ongoing national prospective observational multi-arm multicenter cohort study on SARS-CoV-2 vaccinations in patients with IMIDs using immunosuppressive therapies. This study was approved by the medical ethical committee, and all participants provided signed informed consent (NL74974.018.20, EudraCT 2021-001102-30, Dutch Trial Register Trial ID: NL8900). Details on full study design and methodology have previously been described [6]. Participants with preceding COVID-19 infections were actively recruited for this study. We included participants who completed at least three out of seven daily AE questionnaires per vaccination. Participants vaccinated with Ad.26.COV2.S (Janssen) were excluded for this analysis. Additionally, we recruited healthy controls. Participants were sent daily electronic questionnaires for the first 7 days after each vaccination on AEs to record presence and severity (graded from 0 to 3; with grades higher than 1 indicating interference with daily life) of local and systemic AEs (Additional file 1). Additional questionnaires were sent to record potential allergic reactions, hospital admissions, other severe clinical events and a preceding SARS-CoV-2 infection before the first vaccination (defined as a positive SARS-CoV-2 PCR). Hospital admissions and other severe clinical events were reviewed by the study team to determine the relation to vaccination. We calculated the proportion of clinically relevant adverse events (rAE) which was defined as the occurrence of any systemic AE higher than grade 1 or any systemic AE of grade 1 lasting more than 2 days [5].

Based on the full data set, we constructed a logistic mixed-effects model to calculate the adjusted relative risk (aRR) for a first and third vaccination (versus a second vaccination), for BNT162b2 (Pfizer/BioNTech) and ChAdOx1 nCoV-19 (AstraZeneca) vaccine (versus CX-024414, Moderna), for having an IMID (versus healthy controls) and for a preceding SARS-CoV-2 infection on the occurrence of relevant systemic adverse events. Additionally, sex and age (stratified into <50 or ≥50), which are known risk factors, were included. aRRs are presented with associated 95% CI. Analyses were performed using R (version 4.1.0). This analysis was not a primary endpoint of the full study so no sample size calculation was performed. This study was supported by ZonMw (The Netherlands Organization for Health Research and Development). The sponsor had no role in the design, analyses or reporting of the study.

## Results

**Fig. 1:**
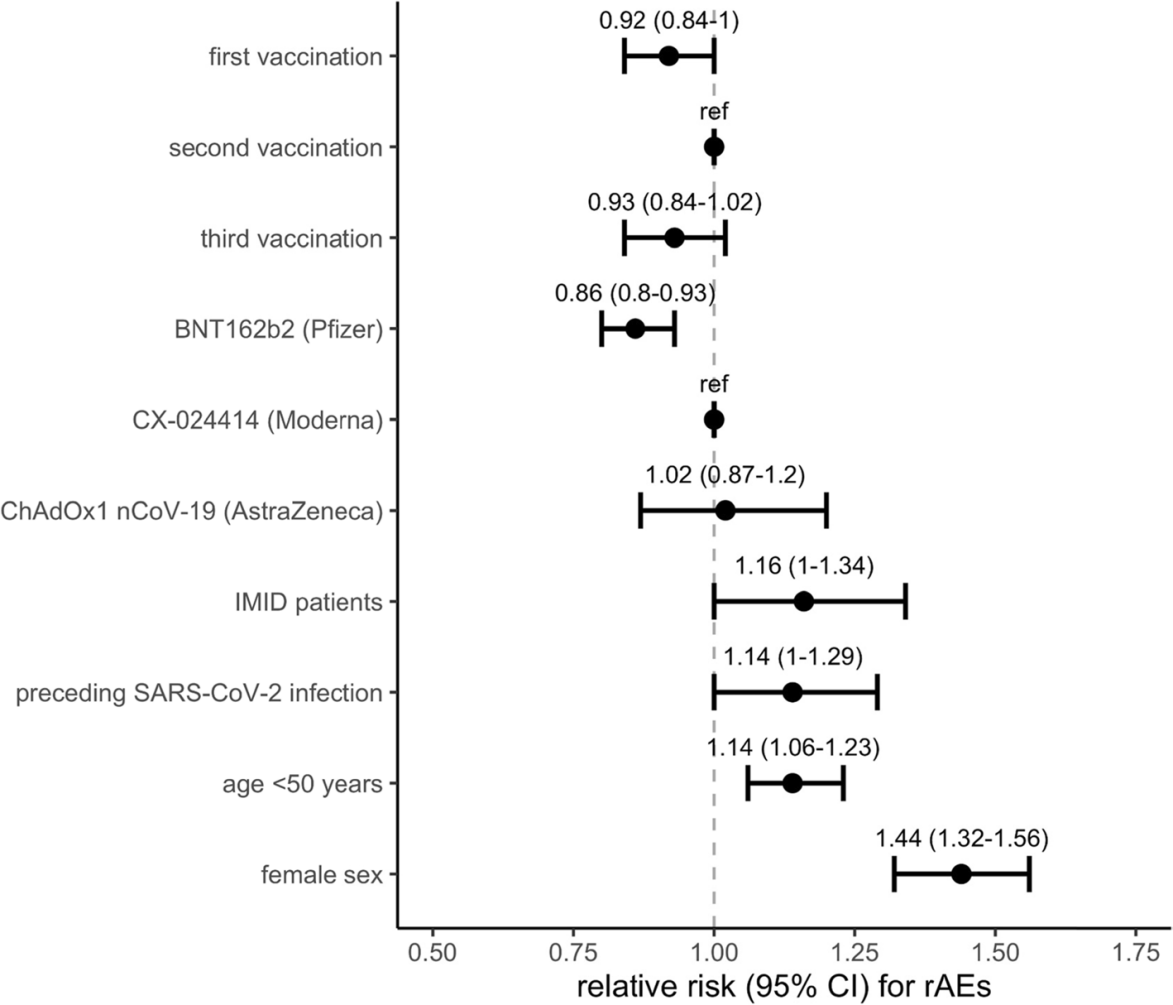
Relative risks for adverse events after SARS-CoV-2 vaccination. Figure showing the adjusted relative risk, including 95% confidence interval, for relevant systemic adverse events after first and third vaccination versus second vaccination, IMID versus no IMID, BNT162b2 and ChAdOx1 nCoV-19 vaccine versus CX-024414, IMID patients versus healthy controls, preceding SARS-CoV-2 infection before first vaccination versus no preceding SARS-CoV-2 infection, age below 50 years versus age above 50 and female versus male sex

As of January 1, 2022, 2259 participants (*N*: 2081 patients and *N*: 178 controls) were included for this analysis (Additional file 2: Fig. S1). Demographics are shown in Additional file 2: Table S1. The overall response rate was 88%; participants who did not complete sufficient questionnaires were younger (mean age 44.1 years; SD 14.9; *p*<0.01) and more frequently males (136/309; 44%; *p*: 0.02). In total, 5207 vaccinations were recorded for primary analysis (1737 first, 1992 second and 1478 third vaccinations). Table 1 displays the percentages of rAEs for patients and controls that varied between 56.4% (95% CI 53.7–58.9) in SARS-CoV-2 naïve IMID patients after the first vaccination and 70.0% (95% CI 61.2–77.6) in IMID patients with preceding COVID-19 infection after the second vaccination. Additional file 2: Fig. S2 shows the proportion and severity of each of the systemic AEs. Fatigue, muscle sore and headache were reported most frequently in all groups. Chills and fever were reported mostly by participants with a preceding SARS-CoV-2 infection. Overall, grade 3 systemic AEs were reported by 7.5% (6.3–8.9; 123/1646) of patients after the first vaccination, by 12.1% (10.7–13.7; 227/1870) after the second vaccination and by 9.5% (8.1–11.2, 137/1437) after the third vaccination. Systemic AEs were similar across the three largest disease groups except for joint pain which was reported mostly by patients with rheumatoid arthritis (Additional file 2: Fig. S3). Ongoing systemic AEs higher than grade 1 were reported by respectively 7.6%, 7.4% and 6.8% of patients at 7 days after their first, second or third vaccination (Table 1). Allergic symptoms after vaccination were reported by 14 patients (0.67%; 95% CI 0.4–1.2). Hospital admissions due to an adverse event were reported by four patients (0.19%; 95% CI 0.06–0.5); one because of an allergic reaction, one patient with epilepsy because of a seizure and two patients with acute cardiovascular disease. In IMID patients, a third vaccination was not associated with an increased risk on adverse events when compared to the second vaccination (aRR 0.93, 95% CI 0.84–1.02) (Fig. 1). A first vaccination was on the other hand associated with lower risk on rAEs (aRR 0.92, 95% CI 0.84–0.99). Furthermore, a preceding SARS-CoV-2 infection before vaccination (aRR 1.14, 95% CI 1.01–1.29) and having an IMID (aRR 1.16, 95% CI 1.01–1.34) were associated with an increased risk of rAEs (Fig. 1). Moreover, female sex (aRR 1.43, 95% CI 1.32–1.56) and age below 50 (aRR 1.14, 95% CI 1.06–1.23) were associated with an increased risk of rAEs. BNT162b2 vaccine was associated with a lower risk on adverse events (aRR 0.86, 95% CI 0.80–0.93), while the ChAdOx1 nCoV-19 vaccine showed similar adverse events (aRR 1.02, 95% CI 0.87–1.20) when compared to CX-024414.

**Table 1:** Relevant adverse event rates in patients with immune-mediated diseases and controls after SARS-CoV-2 vaccination. Table showing percentages of relevant adverse events (rAEs; defined as any systemic AE higher than grade 1 or any systemic AE of grade 1 lasting more than 2 days), systemic AEs at day 7 following vaccination and local AEs higher than grade 1. Percentages are shown with their 95% confidence interval (95% CI) and separately for patients and controls, first, second and third vaccination and individuals with or without a preceding SARS-CoV-2 infection

	Patients	Controls
**First vaccination**		
rAEs	57.3 (54.8–59.8902/1574)	49.7 (41.8–57.6; 81/163)
rAEs, SARS-CoV-2 naive	56.4 (53.7–58.9;807/1432)	45.9 (36.9–55.1; 56/122)
rAEs, with preceding SARS-CoV-2	66.9 (58.4–74.4;95/142)	61.0 (44.5–75.4; 25/41)
Systemic AE>1 at day 7^a^	7.6 (6.3–9.1; 112/1481)	6.3 (3.2–11.6; 10/159)
Local AE>1^a^	15.6 (14.8–18.4; 275/1664)	18.8 (13.4–25.7; 32/170)
**Second vaccination**		
rAEs	61.5 (59.2–63.7;1129/1836)	59.0 (50.8–66.7; 92/156)
rAEs, SARS-CoV-2 naive	60.8 (58.5–63.2; 1038/1706)	58.3 (49.2–66.8; 74/127)
rAEs, with preceding SARS-CoV-2	70.0 (61.2–77.6; 91/130)	62.1 (42.4–78.7; 18/29)
Systemic AE>1 at day 7^a^	7.4 (6.2–8.7; 121/1647)	4.1 (1.7–9.1; 6/146)
Any local AE>1^a^	21.4 (19.6–23.3; 400/1870)	27.2 (20.6–35.0; 43/158)
**Third vaccination**		
rAEs	58.0 (55.3–60.6; 801/1381)	51.5 (41.2–61.7; 50/97)
Systemic AE>1 at day 7^a^	6.8 (5.4–8.3; 83/1229)	2.2 (0.4–8.4; 2/92)
Any local AE>1^a^	19.6 (17.6–21.7; 281/1437)	12.9 (7.3–21.4; 13/101)

Data are shown in percentage (95% CI; *n*/*N*)

^a^For the secondary analyses of systemic rAEs at day 7 and local AEs, in total 5454 vaccination moments were included (as compared to 5207 vaccinations observed for the primary analysis)

## Discussion

We identified that a third vaccination was not associated with an increased risk of short-term AEs in IMID patients. Both having an IMID and a preceding SARS-CoV-2 infection were modest risk factors for AEs. Most AEs are resolved within 7 days; allergic reactions and hospital admissions were uncommon.

We found that the risk of adverse events modestly increased from the first to second vaccination but did not increase further after a third vaccination; this suggests that repeated vaccination therefore does not lead to incremental adverse events. Our findings may be interpreted as reassuring for short-term safety for third and potentially more SARS-CoV-2 vaccinations in IMID patients. We identified also a preceding SARS-CoV-2 infection as an independent risk factor, whereas other smaller studies did not report increased AEs after a second vaccination in individuals with a preceding SARS-CoV-2 infection [5, 7]. Similar to earlier studies, we confirm that female sex, younger age and the CX-024414 vaccine are risk factors for AEs, when compared to BNT162b2 [8–10]. In addition, we found that IMID patients may be slightly more at risk for developing AEs after SARS-CoV-2 vaccination compared to controls.

Despite the large sample size with selected patients and controls and a high overall response rate, the self-reporting character of this study could be considered as a limitation. In addition, we only accounted for SARS-CoV-2 infections before the first vaccination in our analysis. Furthermore, we found allergic reactions and hospital admission to be uncommon in IMID patients, but this study is not large enough to detect rare (serious) adverse events. Lastly, most participants received mRNA vaccines; hence, our results cannot be translated to other vaccines.

## Conclusion

A third SARS-CoV-2 vaccination was not associated with an increased risk on short-term AEs in patients with IMIDs when compared to a second vaccination.

## Supplementary Information

**Additional file 1.** Adverse events questionnaire.

**Additional file 2: Table S1.** Demographics. **Figure S1.** Study flowchart. **Figure S2.** Adverse events in patients with IMIDs after SARS-CoV-2 vaccination. **Figure S3.** Adverse events after SARS-CoV-2 vaccination per IMID.

## Data Availability

The datasets used and analysed during the current study are available from the corresponding author on reasonable request.

## Abbreviations

ARR: Adjusted relative risk
AE: Adverse event
CI: Confidence interval
IMID: Immune-mediated inflammatory disease
rAE: Relevant adverse event
