# Supplementary material for: Risk factors associated with short-term adverse events after SARS-CoV-2 vaccination in patients with immune-mediated inflammatory diseases
Source: BMC Med. 2022 Mar 2;20:100. doi: 10.1186/s12916-022-02310-7 (PMC8889379; doi:10.1186/s12916-022-02310-7)
Supplement: Supplementary file 1 — Additional file 1. Adverse events questionnaire. [file 12916_2022_2310_MOESM1_ESM.pdf]

Additional file 1: Adverse events questionnaire

Did you experience the symptom below directly after or in the hours following your vaccination?

1. Did you experienced an allergic reaction after your vaccination?
   - o Yes
   - o No

   If yes:
   1.1 What kind of allergic reaction?
   - o Hives (urticaria)
   - o Swelling face/mouth/tong (angio-oedema)
   - o Fainting (syncope)
   - o Shortness of breath
   - o Vomiting
   - o Diarrhea
   - o Stomach ache
   - o Shock (life-threatening situation which needed acute treatment)

   1.2 Did you receive treatment from a physician?
   - o Yes
   - o No

Did you experience any of the symptoms below in the last 24 hours?

2. Redness at vaccination site:
   - o No
   - o Yes, 2.5 - 5.0 cm in diameters
   - o Yes, 5.1 – 10.0 cm in diameters
   - o Yes, more than 10 cm in diameters

3. Swelling at the vaccination site:
   - o No
   - o Yes, 2.5 - 5.0 cm in diameters
   - o Yes, 5.1 – 10.0 cm in diameters
   - o Yes, more than 10 cm in diameters

4. Pain at the vaccination site:
   - o No
   - o A little, but it did not hinder at my daily activities
   - o Yes, it hindered me at my daily activities or I needed more than one painkiller (but no heavy painkillers such as morphine or oxycodone)
   - o A lot, I could not perform my daily activities or I needed a heavy painkiller such as morphine of oxycodone

5. Fever:
   - o No
   - o Between 38.0 - 38.4 degrees Celsius

- o Between 38.5 - 38.9 degrees Celsius
- o 39.0 degrees Celsius or above

6. Chills:
   - o No
   - o A little, but it did not hinder at my daily activities
   - o Yes, it hindered me at my daily activities
   - o A lot, I could not perform my daily activities

7. Fatigue:
   - o No
   - o A little, but it did not hinder at my daily activities
   - o Yes, it hindered me at my daily activities
   - o A lot, I could not perform my daily activities

8. Headache:
   - o No
   - o A little, but it did not hinder at my daily activities
   - o Yes, it hindered me at my daily activities
   - o A lot, I could not perform my daily activities

9. Muscular pain:
   - o No
   - o A little, but it did not hinder at my daily activities
   - o Yes, it hindered me at my daily activities
   - o A lot, I could not perform my daily activities

10. Joint pain (for example a knee or wrist):
    - o No
    - o A little, but it did not hinder at my daily activities
    - o Yes, it hindered me at my daily activities
    - o A lot, I could not perform my daily activities

11. Nausea:
    - o No
    - o A little, but it did not hinder at my daily activities
    - o Yes, it hindered me at my daily activities
    - o A lot, I could not perform my daily activities

12. Did you contact a physician in the last 24 hours for any of the symptoms mentioned above?
    - o Yes
    - o No
