# Supplementary material for: Risk factors associated with short-term adverse events after SARS-CoV-2 vaccination in patients with immune-mediated inflammatory diseases
Source: BMC Med. 2022 Mar 2;20:100. doi: 10.1186/s12916-022-02310-7 (PMC8889379; doi:10.1186/s12916-022-02310-7)
Supplement: Supplementary file 2 — Additional file 2: Table S1. Demographics. Figure S1. Study flowchart. Figure S2. Adverse events in patients with IMIDs after SARS-CoV-2 vaccination. Figure S3. Adverse events after SARS-CoV-2 vaccination per IMID. [file 12916_2022_2310_MOESM2_ESM.docx]

**Additional file 2: Supplementary table S1 and figures S1-S3**

*Supplementary table 1: demographics*

|  | | **Patients (N:2081)** | **Controls (N:178)** |
| --- | --- | --- | --- |
| **Age**, mean (SD) | | 50 (14) | 48 (11) |
| **Female sex**, N (%) | | 1306 (62.8) | 117 (65.7) |
| **Vaccine type first and second vaccination**, N (%): | |  |  |
|  | CX-024414 (Moderna) | 612 (29.4) | 105 (60.3) |
|  | BNT162b2 (Pfizer/BioNtech) | 1209 (58.1) | 65 (37.4) |
|  | mRNA vaccine, not further specified | 27 (1.3) | 2 (1.2) |
|  | ChAdOx1 nCoV-19 (AstraZeneca) | 233 (11.2) | 2 (1.2) |
|  | Missing | 0 (0) | 4 (2.2) |
| **Vaccine type third vaccination,** N (%): | |  |  |
| CX-024414 (Moderna) | | 611 (44.2) | 42 (43.3) |
| BNT162b2 (Pfizer/BioNtech) | | 478 (34.6) | 40 (41.2) |
| mRNA vaccine, not further specified | | 292 (21.1) | 15 (15.5) |
| **COVID-19 infection prior to vaccination**, N (%) | | 173 (8.3) | 43 (24.2) |
| **Immune-mediated inflammatory disorders**, N (%): | |  |  |
|  | **Dermatological** |  |  |
|  | atopic dermatitis | 115 (5.5) |  |
|  | other dermatologic conditions^a^ | 177 (8.5) |  |
|  | **Neurological** |  |  |
|  | multiple sclerosis and neuromyelitis optica^b^ | 343 (16.5) |  |
|  | myasthenia gravis | 123 (5.9) |  |
|  | inflammatory neuropathies and myopathies^c^ | 153 (7.4) |  |
|  | **Rheumatological** |  |  |
|  | rheumatoid arthritis | 266 (12.8) |  |
|  | spondyloarthritis | 114 (5.5) |  |
|  | SLE, and Sjogren’s disease | 158 (7.6) |  |
|  | vasculitis^d^ | 72 (3.5) |  |
|  | other rheumatological diseases | 28 (1.3) |  |
|  | **Gastro-enterological** |  |  |
|  | Crohn’s disease | 302 (14.5) |  |
|  | ulcerative colitis | 178 (8.6) |  |
|  | other gastro-enterological conditions^e^ | 46 (2.2) |  |
|  | **Other immune-mediated inflammatory disorder** | 6 (0.3) |  |

a: including vitiligo, pemphigus, psoriasis; b: 5 patients with neuromyelitis optica; c: including chronic inflammatory demyelinating polyneuropathy, multifocal motor neuropathy, myositis; d: including giant-cell arteritis, small vessel vasculitis and other forms of vasculitis; e: including autoimmune hepatitis and autoimmune sclerosing cholangitis.

*
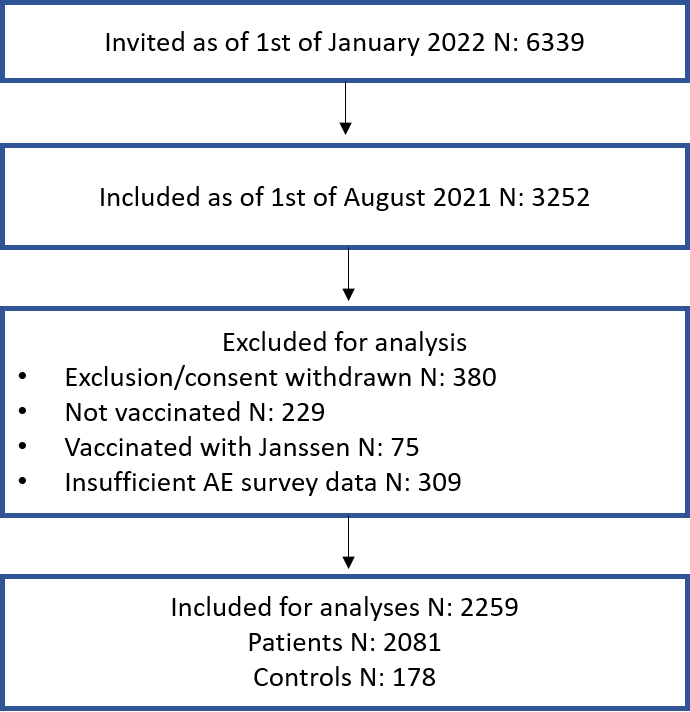
*

*Supplementary figure 1: study flowchart*


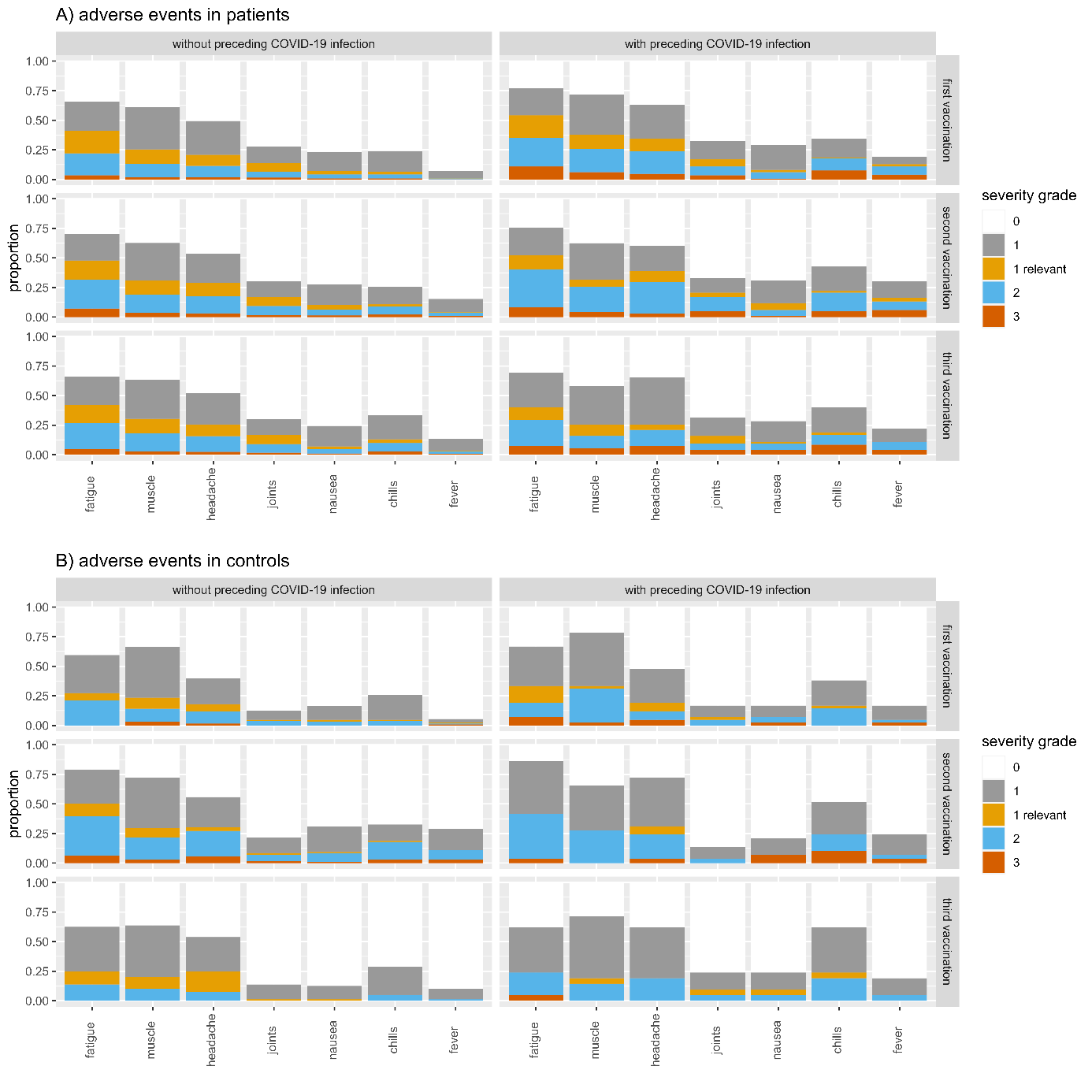


*Supplementary figure 2: Adverse events in patients with IMIDs after SARS-CoV-2 vaccination*

Figure showing proportions of the highest reported severity grade by individuals for any of systemic AEs separately for patients (A) and controls (B), first, second and third vaccinations and individuals with or without a preceding SARS-CoV-2 infection during the first 7 days after vaccination. Severity grade 0 indicates no AE, grade 1 indicates no interference in daily life, grade “1 relevant” indicates a grade 1 category lasting more than 2 days, grade 2 indicates some hindrance at daily activities, and grade 3 indicates such hindrance that daily activities could not be performed.


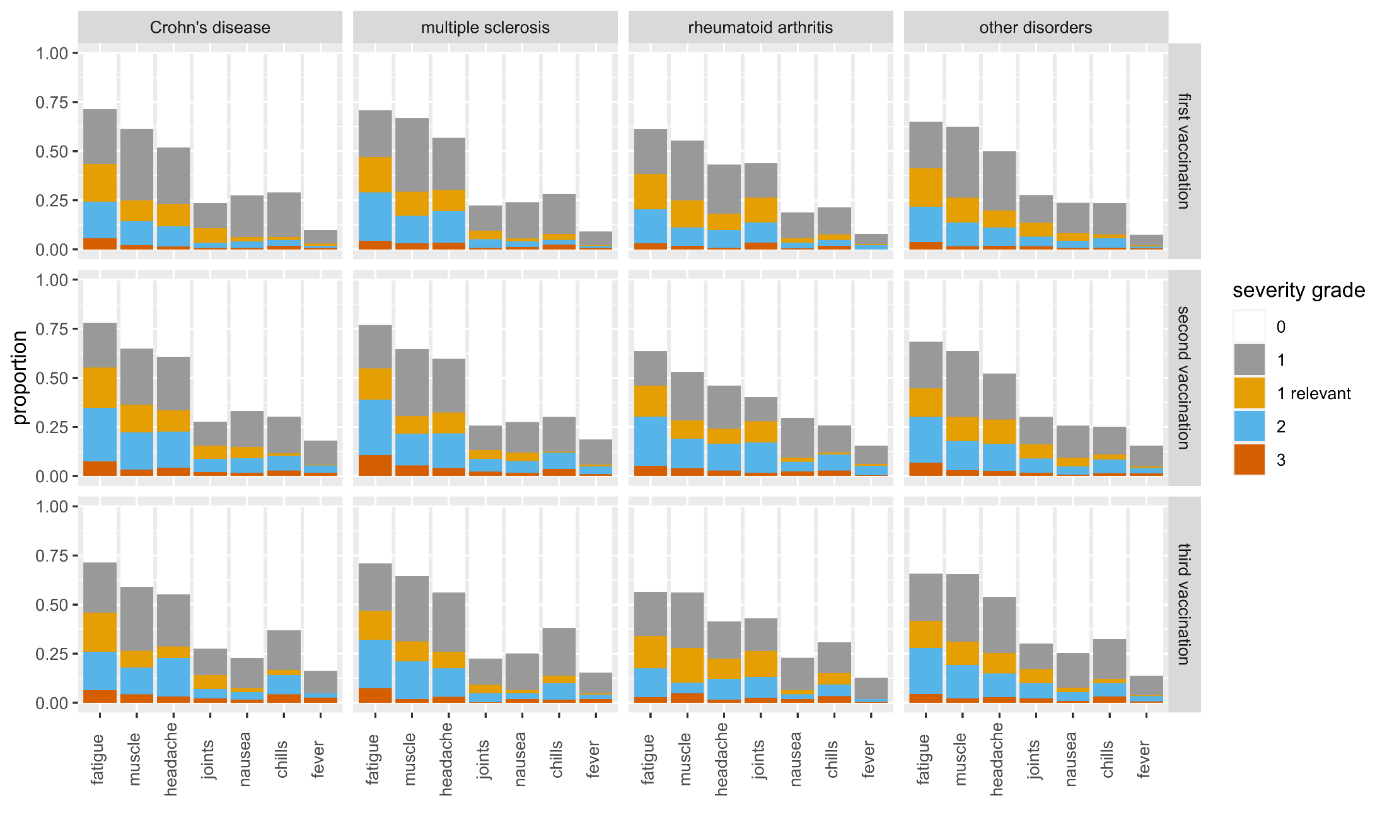


*Supplementary figure 3: Adverse events after SARS-CoV-2 vaccination per IMID*

Figure showing proportions of the highest reported severity grade by individuals for any of systemic adverse events for three largest disease groups and other disorders, separately for first, second and third vaccinations. Severity grade 0 indicates no AE, grade 1 indicates no interference in daily life, grade “1 relevant” indicates a grade 1 category lasting more than 2 days, grade 2 indicates some hindrance at daily activities, and grade 3 indicates such hindrance that daily activities could not be performed.
